# Short-term energy drink consumption influences plasma glucose, apolipoprotein B, body mass index and pulse rate among students

**DOI:** 10.15190/d.2022.18

**Published:** 2022-12-31

**Authors:** Munachimso Mariasonia Iheanacho, Rosemary Adamma Analike, Samuel Chukwuemeka Meludu, Emmanuel Chukwuemeka Ogbodo, Christian Ejike Onah

**Affiliations:** ^1^Department of Medical Laboratory Science, Faculty of Health Sciences and Technology, Nnamdi Azikiwe University, Awka, Nigeria; ^2^Department of Chemical Pathology, Faculty of Medicine, Nnamdi Azikiwe University, Awka, Nigeria; ^3^Department of Human Biochemistry, Faculty of Basic Medical Sciences, Nnamdi Azikiwe University, Awka, Nigeria

**Keywords:** Energy drink, fearless energy drink, predator energy drink, BMI, blood pressure, plasma glucose, apolipoproteins, triglyceride level.

## Abstract

OBJECTIVE: Energy drinks are becoming more popular every year, particularly among young adults such as college students, despite evidence that they have harmful health effects. The effect of energy drink consumption on plasma glucose, serum apolipoproteins, and triglyceride levels in students was investigated. METHODS: In order to test this, we chose two representative types of energy drinks in Nigeria, namely fearless and predator. These energy drinks are brand names of non-alcoholic beverages aimed to provide energy. 30 students, apparently healthy male human subjects aged 18 to 30 years who gave informed consent to the research work were randomly selected and divided into two groups: Group A (fearless energy drink consumers, n=15) and Group B (predator energy drink consumers, n=15). RESULTS: The results demonstrated significant reductions in pulse rate (86.00±41.32 vs. 78.87±27.72; p=0.03) and BMI (21.41±1.93 vs. 21.7±12.02; p=0.00) as compared to baseline values after one month of “fearless energy drink” consumption. Plasma glucose levels were significantly higher (97.53±10.62 vs. 88.80±11.33; p=0.01) and Apo B levels were significantly lower (21.41±1.93 vs. 21.71±2.02; p=0.00) following two weeks of fearless energy drink consumption than in baseline. In addition, BMI and Apo B levels were significantly lower after two weeks of predator energy drink consumption, but plasma glucose levels were significantly higher after two weeks and one month of predator energy drink consumption, respectively (p<0.05). SBP, DBP, TG and Apo A levels did not differ significantly in both fearless and predator energy drink consumers at baseline and after the study period respectively (p>0.05). CONCLUSION: This study has shown that the consumption of energy drinks causes significant alterations in BMI, pulse rate, plasma glucose and apolipoprotein B levels which may have important clinical consequences for energy drink consumers

## INTRODUCTION

Energy drinks are caffeine-containing soft drinks with one or more additional ingredients, such as taurine, glucuronolactone, inositol, herbal extracts (such as guarana, yerba mate, and ginseng), vitamins (such as riboflavin, niacin, and vitamin B-6), proprietary blends, and amino acids, in addition to caffeine^1^. They were first introduced in Austria in 1987 and are now sold in over 140 countries^2^. Teenagers and young people are particularly fond of energy drinks^3^, due to its ability to increase energy, boost alertness, and encourage wakefulness when engaging in high-intensity physical activity, and it has become one of the most often used substances by athletes and other physical activity enthusiasts^4^. These drinks were first introduced to Nigeria in 1997^5^, despite the fact that they seem to be a recent trend. They have been readily available to the general public ever since. Despite the wide variety of energy drink available, most contain comparable ingredients such as water, sugar, caffeine, non-nutritive stimulants (such as guarana, ginseng, yerba mate, taurine, L-carnitine, D-glucuronolactone, and inositol), and vitamins and minerals (such as B vitamins)^6,7^. Caffeine concentration in energy drinkvaries greatly, ranging from 47 to 80 mg per 8 oz to 207 mg per 2 oz, and comes from a variety of sources^8^. Moderate caffeine consumption (up to 400 mg per day) is usually regarded as safe and even advantageous to adults' health^9^. High fructose corn syrup, sucrose, or artificial sweeteners are also found in excessive concentrations in energy drinks. The amount of sugar in one can of energy drink (500 ml or 16.9 oz) is usually around 54 g^10^. Because of the significant evidence linking added sugar consumption to poor health, many institutions, including the World Health Organization (WHO), have suggested lowering sugar intake^11^.

Guarana is a South American plant extract that contains a considerable amount of caffeine, with one gram of guarana equaling 40 mg of caffeine^12^. It is frequently added as an ingredient in energy drinkfor its stimulatory impact due to its high caffeine content^6^. Ginseng, on the other hand, is an herbal supplement that has been used in East Asia for hundreds of years and is said to provide health advantages such as vasorelaxation, antioxidation, anti-inflammation, and anticancer properties^13^. Yerba mate, like guarana, has high caffeine content (78 mg caffeine per cup) and is also thought to boost antioxidant status, minimize weight gain, and prevent cancer^6,14^. Taurine has been recommended as a treatment for epilepsy, heart failure, cystic fibrosis, and diabetes due to its anti-inflammatory properties^15^. The most frequent B vitamins found in energy drinkare B2 (riboflavin), B3 (niacin), B6 (pyridoxine, pyridoxal, and pyridoxamine), and B12^6^. Other additives such as L-carnitine, D-glucuronolactone, and inositol have a limited amount of research on their content and function, with only a few studies suggesting moderate benefits^13^. Importantly, while some authorities credit a number of health benefits to energy drink consumption, energy drink usage may predispose consumers to and act as a contributory risk factor in the development of cardiovascular illnesses and diabetes mellitus.

Lipoproteins (VLDL, LDL, IDL, HDL, and chylomicrons) are the primary transporters of lipids (cholesterol and free fatty acids) in the body, and there are five different types of lipoproteins in the system: very low, low, intermediate, and high-density lipoproteins (VLDL, LDL, IDL, HDL, and chylomicrons, respectively). High LDL levels, low HDL levels, and in particular a low HDL/LDL ratio, are all well-known risk factors for cardiovascular diseases (CVDs) especially, coronary artery disease^16^. CVDs are a group of disorders of the heart and blood vessels. Apolipoproteins (Apo) are found on the surface of lipoproteins, and they both regulate lipid transit and interact with specific receptors to allow lipid uptake and deposition into tissue, making them important players in cholesterol metabolism^17^. According to the findings of several studies, high ApoB concentrations, low ApoA1 concentrations, and the ApoB/ApoA1ratio may be more accurate predictors of CVD risk than LDL, HDL, and the LDL/HDL ratio^16^.

Young people, caffeine-naive or caffeine-sensitive persons, pregnant women, competitive athletes, and people with underlying cardiovascular disease are all at risk of consequences from energy drink consumption^18^. People who consume energy drinks frequently report feeling ill, restless, or agitated, as well as sleeplessness, tachycardia, and an elevated pulse rate^19^. Despite this, data suggest that in industrialized countries, the rate of patronage is significantly lower than in Nigeria, where the beverages are progressively taking over the market^20^. Young peoples' health can be jeopardized by excessive use of energy drinks and accumulation of the foregoing elements, as well as their reciprocal interactions. In healthy young people, consumption of energy drinks induced a considerable increase in blood glucose and blood pressure, according to Nowak and colleagues^21^. Furthermore, poor glucose disposal and insulin resistance have been reported in the past among people who consume caffeine and caffeinated energy drinks^22^, despite the fact that some other authors have found no significant effect of energy drinks on blood glucose levels^23^. Furthermore, multiple research projects have shown that drinking energy beverages causes considerable changes in lipid profile markers^24,25^.

The consumption of energy drinks is becoming more popular, particularly among young adults and students. This rising trend in energy drink intake among young adult students may have been prompted by their desire to improve their academic grades. This is because most energy drinks contain caffeine and sugar, which enhances energy, alertness, and wakefulness, all of which are necessary for academic work. Caffeine use for a short period of time lowers insulin sensitivity in healthy people and may thereby change glucose homeostasis toward hyperglycemia in the short-term which is crucial to the pathophysiology of diabetes mellitus, a disease whose prevalence is also on the rise in Nigeria^26^. More specifically, raised glucose levels, insulin resistance, and changes in lipid profile levels all lead to an increased risk of cardiovascular disease in susceptible people later in life. It's worth noting that, to our knowledge, this is the first time a study of this kind has been conducted among Nnamdi Azikiwe University students, evaluating the influence of energy drinks on plasma glucose, serum apolipoproteins, and triglyceride levels. As a result, it was critical to carry out the current research.

In order to test the effect of energy drink consumption on plasma glucose, serum apolipoproteins, and triglyceride levels in students, we chose two representative types of energy drinks in Nigeria, namely fearless and predator. These energy drinks are brand names of non-alcoholic beverages aimed to provide energy.

## MATERIALS AND METHODS

### Study design and participant recruitment

This is a case study to see how a particular energy drink affects blood glucose, apolipoproteins, and triglyceride levels in students at the College of Health Sciences in Nnewi, Anambra State, Nigeria. For this study, thirty (30) apparently healthy male volunteers aged eighteen (18) to thirty (30) years were recruited at random and divided into two groups: group A (fearless energy drink consumers) and group B (predator energy drink consumers), each comprising fifteen male subjects. Each individual was given a thorough explanation of the study methodology before agreeing to participate. For a period of two weeks, each subject was also told to avoid caffeine-containing commodities such as tea, chocolate, and cola beverages, fearless and predator energy drinks, and other similar drinks. Following that, group A individuals were given one bottle of fearless energy drink (500 ml) and group B subjects were given one bottle of predator energy drink (400 ml) four times per week (Tuesdays, Thursdays, Saturdays, and Mondays) for a total of twenty-eight days prior to their daily breakfast. Students in Group A were given fearless energy drink, whereas those in Group B were given predator energy drink**. **100 ml offearless energy drink contains : water, carbohydrate (12 g sugars), carbon dioxide, citric acid E 330, flavoring, acidity regulator (sodium citrate E 331), taurine, caffeine (0.031%), inositol, niacin (3 mg), colors (tartrazina E102 and sunset yellow FCF E110), ginseng extract, vitamin B6 (0.3 mg), vitamin B12 (0.3µg), fiber (<0.5 g), protein (<0.5 g), salt (0.02 g), fat (<0.5 g) of which saturates (<0.1 g) and energy value (283 KJ). 400 ml of Predator energy drink contains: carbonated water, sucrose, acids (citric acid, tartaric acid), acidity regulator (sodium citrate), nature identical and artificial pineapple, and carbohydrate (56 g), of which sugars were 54g, sodium (0.2 g), niacin (12.8 mg, 85%), vitamin B6 (1.2mg, 92%), and caffeine (120mg), flavor, inositol (1 mg/100 ml), taurine (100 mg/100 ml), and preservatives (potassium sorbate and sodium benzoate) and negligible fat, saturates, and proteins.

Following an overnight fast, 5 mls of baseline samples (day zero), post research sample I (day 14), and post research sample II (day 29) were collected into fluoride oxalate and plain containers in appropriate volume for estimation of plasma glucose, serum apolipoprotein A-I, apolipoprotein B100, and triglyceride levels.

The participants' age, medical history, and food habits were all collected using a structured questionnaire.

### Study Area

This study was carried out at College of Health Sciences, Nnamdi Azikiwe University, Nnewi Campus, Anambra State, Nigeria.

### Inclusion criteria

The study comprised apparently healthy College of Health Sciences, Nnewi students between the ages of 18 and 30.

### Exclusion criteria

This study excluded smokers, alcoholics, those with pre-existing diseases (diabetes, hypertension, and cardiovascular disease).

### Ethical consideration

The ethical approval for this research was sought and obtained from the Faculty of Health Science and Technology ethics committee prior to the commencement of the study (NAU/FHST/2021/MLS117).

### Informed consent

Prior to the start of the study, the participants were asked to provide written informed consent.

### Collection of samples

After a 10- to 12-hour fast, 5 ml of venous fasting blood was obtained aseptically from each participant via the antecubital vein using a plastic syringe with minimal stasis and placed in a proper proportion in a fluoride oxalate bottle and plain container. The blood sample in the plain tube was allowed to clot and retract before being centrifuged for 10 minutes at 1000 rpm. The serum was separated and used in a routine laboratory manner to determine serum triglyceride, apolipoprotein A, and B levels. Samples that could not be analyzed immediately were frozen.

### Biochemical methods

The glucose oxidase peroxidase method described by^27^ was used to determine plasma glucose using a Randox test kit.

The serum triglyceride level was tested using a Randox test kit and the enzymatic method described by^28^. Apolipoprotein A-I (ApoA1) and Apolipoprotein B (ApoB) levels in human serum were determined using the Tietz method^29^, as reported by Ezeugwunne et al.^30^, utilizing the Biobase test kit from China.

### Anthropometric measurements

Body mass index (BMI) was calculated using the following formula: BMI= weight (kg) / height^2^ (m^2^). A measuring tape was fastened to a piece of wood to determine height, and a manual weighing scale was used to determine weight. BMIs of 25 and 30 kg/m^2^ were used to classify overweight and generalized obesity, respectively.

### Blood pressure reading

Systemic blood pressure was obtained using an OMRON automatic digital blood pressure monitor on the left arm after 10-minute rest using a cuff of appropriate size with the subject in the sitting position. Blood pressure was expressed as systolic and diastolic rate. Hypertension was defined as systolic blood pressure *≥ *140mmHg and/or diastolic blood pressure *≥*90mmHg.

### Statistical Analysis

The results were examined using the Statistical Package for the Social Sciences (SPSS) version 23.0, with the data provided as mean ± standard deviation (SD) and statistically assessed using the paired t-test and Pearson r correlation. The significance of the threshold was established at p<0.05.

## RESULTS

The mean systolic blood pressure (SBP) and diastolic blood pressure (DBP) did not differ significantly when compared between the different groups studied (p>0.05) respectively. Also, the mean pulse rate did not differ significantly between all the groups studied except when compared before fearless energy drink consumption and after one month of fearless energy drink intake which was significantly decreasedafter one month of fearless energy drink intake (86.00±41.32 vs. 78.87±27.72; p=0.03). Furthermore, the mean body mass index was significantly decreased in predator energy drink consumers following two weeks intake when compared to their value before predator energy drink intake (21.48±3.06 vs. 22.03±3.04; p=0.01). Again, there was a significant decrease in the mean BMI after one-month intake of fearless energy drink than before the intake of fearless energy drink (21.41±1.93 vs. 21.71±2.02; p=0.00). Meanwhile, both mean height and weight of the subjects studied did not differ significantly when compared between the groups studied (p>0.05) respectively (Table 1).

**Table 1:** Comparison of mean ± SD of SBP, DBP, pulse and BMI of subjects studied *Statistically significant at p<0.05. key: 1 = before consumption of fearless energy drink; 2 = before consumption of predator energy drink; 3 = after 2 weeks consumption of fearless energy drink; 4 = after 2 weeks consumption of predator energy drink; 5 = after 1 month consumption of fearless energy drink; 6 = after 1 month consumption of predator energy drink.

Variables	SBP (mmHg)	DBP (mmHg)	Pulse rate (beat/minute)	BMI (Kg/m2)	Height (cm)	Weight (Kg)
1. Before consumption: fearless energy drink consumers (n=15)	125.73±9.95	75.73±9.08	86.00±41.32	21.71±2.02	178.09±8.81	69.20±10.07
2. Before consumption: predator energy drink consumers (n=15)	122.07±9.76	73.53±7.70	75.13±20.52	22.03±3.04	178.68±6.85	70.27±9.79
3. After 2 weeks consumption: fearless energy drink consumers (n=15)	124.93±10.09	75.60±8.62	74.53±10.27	22.15±3.34	178.03±9.89	68.50±10.12
4. After 2 weeks consumption: predator energy drink consumers (n=15)	122.27±9.12	74.20±9.02	75.20±12.41	21.48±3.06	178.60±9.85	68.53±9.92
5. After 1 month consumption: Fearless energy drink consumers (n=15)	124.73±6.57	77.53±5.88	78.87±27.72	21.41±1.93	178.11±7.90	68.57±9.88
6. After 1 month consumption: Predator energy drink consumers ( n=15)	119.80±8.03	75.87±5.44	76.53±9.30	22.05±3.31	178.70±7.85	68.60±9.85
1 V2 (p-value)	0.32	0.40	0.37	0.74	0.84	0.77
3 V4 (p-value)	0.45	0.67	0.87	0.13	0.70	0.99
5 V6 (p-value)	0.08	0.43	0.76	0.52	0.81	0.57
1 V4 (p-value)	0.21	0.32	0.81	0.49	0.69	0.73
2 V4 (p-value)	0.93	0.78	0.99	0.01*	0.92	0.68
1 V5 (p-value)	0.21	0.20	0.03*	0.00*	0.63	0.79
2 V6 (p-value)	0.34	0.22	0.73	0.92	0.59	0.61
1 V3 (p-value)	0.74	0.95	0.36	0.53	0.80	0.80
1 V6 (p-value)	0.80	0.90	0.41	0.61	0.55	0.71
3 V4 (p-value)	0.78	0.83	0.37	0.50	0.60	0.54
3 V6 (p-value)	0.70	0.45	0.43	0.06	0.90	0.67
3 V5 (p-value)	0.93	0.35	0.59	0.31	0.78	0.50
4 V6 (p-value)	0.87	0.29	0.44	0.29	0.76	0.61

There was a significant increase in the plasma glucose level in the predator energy drink consumers after two weeks intake when compared to the values obtained before predator energy drink consumption (101.00±8.49 vs. 91.80±7.05; p=0.002). Also, there was a significant increase in the mean plasma glucose level in the predator energy drink consumers after one-month intake than before the intake of predator energy drink (95.07±7.81 vs. 91.80±7.05; 0.03). Again, there was a significant increase in the mean plasma glucose level in the fearless energy drink consumers after 2 weeks intake than before the intake of fearless energy drink (97.53±10.62 vs. 88.80±11.33; 0.01). There were no significant differences (p>0.05) observed in the mean serum triglyceride and APO A-I levels in both fearless and predator energy drink consumers when compared between the groups studied respectively. However, there was a significant decrease in the mean serum APO B level in predator energy drink consumers 2 weeks after predator energy drink consumption compared to baseline value (0.98±0.17 Vs 1.15±0.16; p=0.03). Also, there was a significant decrease in the mean serum APO B level observed in the fearless energy drink consumers after 2 weeks consumption of fearless energy drink than in baseline (0.88±0.98 Vs 1.24±0.18; p=0.00). Furthermore, there was a significant decrease in the mean serum APO B level observed in the fearless energy drink consumers after 2 weeks consumption of fearless energy drink when compared to the value observed in the predator energy drink consumers after 1 month of predator energy drink consumption (0.88±0.98 Vs 1.00±0.14; p=0.00) (Table 2).

**Table 2:** Comparison of mean ± SD of plasma glucose, triglyceride, Apo A1 and Apo B levels of subjects studied *Statistically significant at p<0.05. key: 1 = before consumption of fearless energy drink; 2 = before consumption of predator energy drink; 3 = after 2 weeks consumption of fearless energy drink; 4 = after 2 weeks consumption of predator energy drink; 5 = after 1 month consumption of fearless energy drink; 6 = after 1 month consumption of predator energy drink.

Variables	Glucose (mmol/L)	Triglyceride (mmol/L)	Apo A1 (g/L)	Apo B (g/L)
Before consumption: fearless energy drink consumers (n=15)	88.80±11.33	0.81±0.15	1.21±0.20	1.24±0.18
Before consumption: predator energy drink consumers (n=15)	91.80±7.05	0.92±0.27	1.27±0.06	1.15±0.16
After 2 weeks consumption: fearless energy drink consumers (n=15)	97.53±10.62	0.78±0.27	1.26±0.11	0.88±0.98
After 2 weeks consumption:predator energy drink consumers (n=15)	101.00±8.49	0.89±0.35	1.28±0.06	0.98±0.17
After 1 month consumption: fearless energy drink consumers (n=15)	90.73±9.31	0.74±0.15	1.24±0.15	0.90±0.22
After 1 month consumption:predator energy drink consumers (n=15)	95.07±7.81	0.77±0.20	1.26±0.11	1.00±0.14
1 V2 (p-value)	0.39	0.20	0.34	0.35
3 V4 (p-value)	0.33	0.26	0.54	0.14
5 V6 (p-value)	0.18	0.52	0.36	1.50
1 V4 (p-value)	0.28	0.28	0.44	0.90
2 V4 (p-value)	0.002*	0.80	0.63	0.03*
1 V5 (p-value)	0.30	0.86	0.75	0.66
2 V6 (p-value)	0.03*	0.19	0.56	0.72
1 V3 (p-value)	0.01*	0.62	0.49	0.00*
1 V6 (p-value)	0.23	0.58	0.62	0.42
3 V4 (p-value)	0.59	0.51	0.50	0.39
3 V6 (p-value)	0.56	0.20	0.72	0.00*
3 V5 (p-value)	0.93	0.36	0.64	0.83
4 V6 (p-value)	0.61	0.39	0.59	0.57

Significant positive correlations were observed in the following parameters studied: pulse_1_ vs. pulse_3_, weight_1_ vs. weight_3_, BMI_1_ vs. BMI_1_, SBP_1_ vs. SBP_2_, Weight_1_ vs. weight_2_, BMI_1_ vs. BMI_2_, SBP_2_ vs. SBP_3_, Weight_2_ vs. Weight_3_ and BMI_2_ vs. BMI_3_ respectively (Table 3).

**Table 3:** Levels of association between parameters studied Statistically significant at p<0.05. key: 1= before consumption of fearless energy drink; 2= before consumption of predator energy drink; 3= after 2 weeks consumption of fearless energy drink.

Parameters	Subjects	Correlation Pearson r coefficient	f-value	p-value
Pulse1 Vs pulse3	n=15	0.57	0.026	P<0.05
Weight1 Vs weight3	n=15	0.98	0.000	P<0.05
BMI1 Vs BMI1	n=15	0.96	0.000	P<0.05
SBP1 Vs SBP2	n=15	0.57	0.027	P<0.05
DBP1 Vs DBP2	n=15	0.60	0.019	P<0.05
Weight1 Vs weight2	n=15	0.99	0.000	P<0.05
SBP2 Vs SBP3	n=15	0.54	0.039	P<0.05
BMI2 Vs BMI3	n=15	0.59	0.020	P<0.05
Weight2 Vs weight3	n=15	0.97	0.000	P<0.05
BMI1 Vs BMI2	n=15	0.62	0.014	P<0.05

## DISCUSSION

Energy drinks are becoming more popular every year among a wide range of consumers, including college students, athletes, amateur competitors, and even those suffering from work-related fatigue, despite evidence suggesting that a significant number of people who consume energy drinks suffer from morbidity and/or mortality as a result of their consumption^18^. As a result, the current study examined the effects of short-term energy drink intake (fearless and predator energy drinks) on plasma glucose, serum apolipoproteins, and triglyceride levels among Nnamdi Azikiwe University students. The mean systolic blood pressure (SBP) and diastolic blood pressure (DBP) of the subjects studied before energy drink consumption were not significantly different from their mean values after two weeks of energy drink consumption and after one month of energy drink consumption in both fearless and predator energy drink consumers, respectively. In a placebo-controlled research, Ragsdale *et al*. found no change in blood pressure over a 2-hour period after drinking 250 ml of energy drink^31^. In addition, Hajsadeghi*et al*. found that a 250 ml dose of energy drink (80 mg of caffeine) had no statistically significant effect on blood pressure (SBP and DBP) in 44 healthy people evaluated 0.5, 2 and 4 hours after consumption^32^. In contrast to the current study, earlier research has found significantly higher SBP and DBP in participants after consuming several brands of energy drinks than in the control group^33,34^. However, in their study, Nowak *et al*. noted that acute use of energy drinks resulted in a large increase in DBP, with no significant impact on SBP^21^, which partly aligns with our findings. This current finding could be due to differences in the amounts of various substances contained in different brands of energy drinks, with an emphasis on caffeine levels. Caffeine use has traditionally been linked to hemodynamic alterations^35^. Caffeine ingestion can raise plasma renin, catecholamines, and dopamine levels in the short term. These chemicals excite the central nervous system, causing blood pressure and heart rate to rise^6^. Furthermore, synergistic effects of different components of different energy drink brands may impact blood pressure in a variety of ways, possibly depending on dosage and length of usage. Specifically, taurine has been found in earlier research to have a hypotensive impact^36^. After one month of use, neither fearless energy drink nor predator energy drink had any significant influence on the blood pressures of their respective consumers, according to this study. In this study, the mean pulse rate of fearless energy drink drinkers was considerably lower after a one-month drinking period when compared to the value before consumption. This is consistent with the findings of Hajsadeghiet al., who observed a statistically significant decrease in heart rate 4 hours after consuming 250 mL of energy drink^32^. In contrast to this finding, some authorities reported significant increases in pulse rate after energy drink consumption compared to control group^18^, while others reported no significant changes in pulse rate after energy drink consumption compared to before energy drink consumption and/or control^21,37^, which corroborate well with the current findings among predator energy drink consumers who revealed no significant changes in pulse rate after energy drink consumption. The considerable decrease in pulse rate after one month of continuous energy drink intake could be linked to the taurine content of the energy drink, which has been found to have cardiovascular health benefits^38^. When compared to their baseline, predator energy drink consumers had a considerably lower mean body mass index after two weeks of consumption. In addition, when compared to their baseline value, the mean BMI after one month of energy drink consumption was much lower. The body mass index (BMI=weight (kg)/height (m^2^)) remains the most widely used index of weight status in adults^39^. It is a simple index of weight-for-height that is commonly used to classify overweight and obesity in adults, with normal weight defined as a BMI of 18.5 to 24.9 kg m^-2^; overweight defined as a BMI of 25.0 to 29.9 kg m^-2^; and obesity defined as a BMI of >30.0 kg/m^-240^. The majority of the present participants have a BMI of between 18.5 and 24.9 kg m^-2^. This result could be explained by the fact that the participants in this study were students who are constantly engaged in physical and mental activities that lead to the burning of excess calories. Otherwise, it has been proven that a high-sugar diet is linked to poor physical health, particularly weight gain due to excessive calorie consumption^41^. Energy drinks are heavy in sugar and, as a result, are likely to cause weight gain; unlike equally calorific solid foods, they do not provide satiety. When comparing the data acquired at baseline to the values obtained after two weeks of consumption, the plasma glucose level in the predator energy drink consumers increased significantly. In addition, after one month of consumption, the mean plasma glucose level of predator energy drink consumers was significantly higher than before consumption. When compared to prior fearless energy drinks consumption, there was a substantial increase in the mean plasma glucose level in fearless energy drinks users after two weeks. The increased sugar content seen in energy drinks has amplified this mean value. Nowak and colleagues found that drinking energy drinks induced a significant increase in blood glucose levels in healthy young persons^21^, which is consistent with our findings. Ihim*et al*. previously documented significantly higher plasma glucose levels in Nnamdi Azikiwe University students who consumed coffee, which they attributed to the caffeine content of coffee^42^, which supports the current data. As a result, individuals at risk of diabetes mellitus and those who are already diabetic should be discouraged from drinking energy drinks, as it is well known that type 2 diabetes mellitus is associated with a hallmark of hyperglycemia^43^, which could be exacerbated further with an increased sugar load. Other researchers, on the other hand, have found little evidence that energy drinks have a significant influence on blood glucose levels^23^. There were no statistically significant variations in mean plasma triglyceride levels in the subjects tested in this study when compared between the different groups studied in both the fearless and predator energy drink consumers, respectively. This could mean that both fearless and predator energy drinks have no effect on triglyceride levels after a short time of use. Serum triglyceride is an essential measure of cardiovascular health and is part of the lipid profile or lipid panel group of assays. Triglyceride levels that are too high have long been linked to an elevated risk of cardiovascular disease^44^. The increasing frequency of cardiovascular risk factors is worsening the burden of cardiovascular diseases in emerging nations like Nigeria^45^, and there is an urgent need to reverse these trends. This influenced the study's selection in part. Furthermore, there were no significant differences in mean blood apolipoprotein A-I levels before and after intake of both fearless and predator energy drinks in this investigation. This suggests that after a brief period of ingestion, fearless and predator energy drinks have little influence on Apo AI levels. To the best of our knowledge, this is the first study of its kind to look at the effects of both fearless and predator energy drinks on apolipoprotein levels in consumers after a one-month period of consumption. Surprisingly, when compared to prior energy drink use, the mean serum Apolipoprotein B (ApoB) level was significantly lower after two weeks of fearless and predator energy drink consumption, respectively. In addition, when compared to the value obtained after one month of predator energy drink use, the mean serum Apo B level after two weeks of fearless energy drink consumption was considerably lower. This suggests that short-term ingestion of both fearless and predator energy drinks aids in the reduction of Apo B levels. This is especially important when it comes to cardiovascular health. Apolipoproteins (apolipoproteins) are proteins that bind to lipoproteins (chylomicrons, VLDL, LDL, IDL, and HDL) and transport lipids^46^.

Apo A, B, C, and E are the four major classes of apolipoproteins. The main protein component of high-density lipoprotein cholesterol is apo A-1 (HDL-C). Along with HDL-C, it plays a vital function in cholesterol metabolism. HDL-major role is to take up cholesterol in tissues and transport it back to the liver for bile excretion. Because cholesterol cannot be digested and used as a source of energy in the human body, the only way for the body to get rid of excess cholesterol once its needs have been met is through bile excretion. HDL is aided in this process by Apo A-1. Apo A-1 also activates lecithin-cholesterol acyltransferase (LCAT), an enzyme found on HDL that esterifies the cholesterol it picks up and renders it lipid-soluble so that it may be sequestered deep inside the HDL particles, preventing the cholesterol esters from being lost again. The liver, together with the HDL particles, can then take up the sequestered cholesterol esters. It has been hypothesized that it is a better predictor of CVD risk than HDL-C^16,47^. ApoB100, on the other hand, is made in the liver and found in VLDL, IDL, and LDL. It triggers lipoprotein endocytosis by binding to the LDL receptor. Each VLDL/IDL/LDL has only one ApoB100. ApoB100 is a lipoprotein marker that identifies atherogenic lipoproteins^^48,49^^.

Finally, there were significant positive relationships between pulse rate and weight before fearless ingestion and two weeks after consumption (p<0.05). Additionally, significant positive associations were found between the SBP, DBP, and BMI before and after two weeks of ingestion of the predator energy drink (p<0.05). This means that increasing one of these characteristics causes the other to increase as well, and vice versa.

## CONCLUSION

Although plasma triglyceride and apolipoprotein A1 levels remained identical before and after one month of fearless and predator energy drink use, this study found significant decreases in body mass index and Apolipoprotein B, as well as significant increases in plasma glucose levels. After one month, the consumption of fearless energy drink resulted in a considerable reduction in pulse rate. As a result, while short-term intake of fearless and predator energy drinks may have good impacts on cardiovascular health, it may have substantial and detrimental implications for diabetics.

### KEY POINTS - Recommendations

**◊** *The public should be educated on the effects of energy drink intake on human health, particularly in the case of people with diabetes*

**◊** *If it is impossible to prevent energy drinks consumption, they should be used iin moderation*

**◊** *More research into the impact of energy drinks on cardiometabolic functions should be conducted in order to better understand and unravel the mechanism behind the current findings presented in this study*
